# Proper Sterol Distribution Is Required for *Candida albicans* Hyphal Formation and Virulence

**DOI:** 10.1534/g3.116.033969

**Published:** 2016-08-31

**Authors:** Paula McCourt, Hsing-Yin Liu, Josie E. Parker, Christina Gallo-Ebert, Melissa Donigan, Adam Bata, Caroline Giordano, Steven L. Kelly, Joseph T. Nickels

**Affiliations:** * The Institute of Metabolic Disorders, Genesis Biotechnology Group, Hamilton, New Jersey 08691; † Centre for Cytochrome P450 Biodiversity, Institute of Life Sciences and School of Medicine, Swansea University Medical School, SA2 8PP, UK; ‡ Invivotek, Genesis Biotechnology Group, Hamilton, New Jersey 08691

**Keywords:** virulence, *Candida*, sterol, hyphae, lipid

## Abstract

*Candida albicans* is an opportunistic fungus responsible for the majority of systemic fungal infections. Multiple factors contribute to *C. albicans* pathogenicity. *C. albicans* strains lacking CaArv1 are avirulent. Arv1 has a conserved Arv1 homology domain (AHD) that has a zinc-binding domain containing two cysteine clusters. Here, we explored the role of the CaAHD and zinc-binding motif in CaArv1-dependent virulence. Overall, we found that the CaAHD was necessary but not sufficient for cells to be virulent, whereas the zinc-binding domain was essential, as *Caarv1/Caarv1* cells expressing the full-length zinc-binding domain mutants, Caarv1^C3S^ and Caarv1^C28S^, were avirulent. Phenotypically, we found a direct correlation between the avirulence of *Caarv1/Caarv1*, *Caarrv1^AHD^*, *Caarv1^C3S^*, and *Caarv1^C28S^* cells and defects in bud site selection, septa formation and localization, and hyphal formation and elongation. Importantly, all avirulent mutant strains lacked the ability to maintain proper sterol distribution. Overall, our results have established the importance of the AHD and zinc-binding domain in fungal invasion, and have correlated an avirulent phenotype with the inability to maintain proper sterol distribution.

*Candida albicans* and *Candida glabrata* are pathogenic fungi responsible for the majority of systemic candidiasis cases (Pfaller 1996; Segal 2005; Spellberg 2008). Both are becoming resistant to multiple antifungal drugs, especially the azole class of drugs, and this contributes to clinical resistance (Cowen 2008; Perlin 2014; Rodrigues *et al.* 2014; Shields *et al.* 2015). Although the sterol biosynthesis pathway has become a “hot spot” for acquiring azole resistance (Asai *et al.* 1999; Denning *et al.* 1997; Sanglard *et al.* 1998; Vermitsky and Edlind 2004; Xu *et al.* 2008), it still may be advantageous to target factors involved in maintaining sterol homeostasis. (Borjihan *et al.* 2009; Henneberry and Sturley 2005; Simova *et al.* 2013; Zhang and Rao 2010). Our hypothesis is that disrupting cellular sterol distribution will lead to avirulence. Thus, cell factors regulating this process represent novel drug targets. We believe that Arv1 may represent such a target.

*Saccharomyces cerevisiae ARV1* (Are1 Are2 required for viability) was identified in a genetic screen looking for recessive alleles toxic to *are1 are2* cells (Tinkelenberg *et al.* 2000). The *S. cerevisiae ARE1* and *ARE2* genes are required for yeast sterol esterification (Yang *et al.* 1996). Cells lacking both are viable, but are unable to esterify sterols, thus accumulate free sterol, while *are1 are2 arv1* cells are not (Zweytick *et al.* 2000). Fungi that express Arv1 include *C. albicans* and *C. glabrata* (Gallo-Ebert *et al.* 2012; Tinkelenberg *et al.* 2000). All fungal Arv1 proteins have a conserved amino-terminal Arv1 homology domain (AHD) that contains a consensus zinc-binding motif [C–xx–C–(∼20)–CxxC] (Figure 1) (Fores *et al.* 2006). The topology of the *S. cerevisiae* Arv1 has been solved. It has three endoplasmic reticular transmembrane-spanning regions, a cytoplasmic-facing AHD, and a single large luminal loop region (Georgiev *et al.* 2013; Villasmil and Nickels 2011). *arv1* cells are hypersusceptible to the ergosterol-binding agent nystatin, suggesting a mislocalization of sterol to the plasma membrane (Tinkelenberg *et al.* 2000). Cells lacking Arv1 accumulate several unknown sterol intermediates, suggesting these cells have defects in sterol synthesis (Kajiwara *et al.* 2008), and they harbor lipid distribution defects, as they cannot polarize phosphatidylinositol 4,5 phosphate (PIP_2_) during yeast mating (Fei *et al.* 2008; Villasmil *et al.* 2011). Mutant cells also have defects in organelle lipid morphology and homeostasis (Georgiev *et al.* 2013; Schechtmans *et al.* 2011), and they are highly sensitive to fatty acid supplementation (Ruggles *et al.* 2014). *arv1* cells are highly susceptible to the depsipeptide phosphatidylserine binding agent papuamide-B, indicating elevated levels in the plasma membrane (Georgiev *et al.* 2013). Thus, there is strong evidence that Arv1 regulates lipid distribution in *S. cerevisiae*.

**Figure 1:**
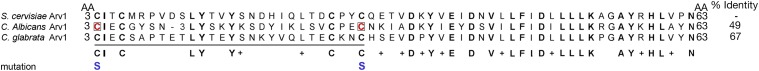
Shown is a schematic representation of AHD motifs from several eukaryotes. The zinc-binding motif is underlined and conserved (+) or identical amino acids (bold) are indicated. Amino acids mutated are in red, and amino acid substitutions are in blue.

*C. albicans Caarv1/Caarv1* cells are hypersusceptible to the polyenes, nystatin and amphotericin B, itraconazole, and lovastatin (Gallo-Ebert *et al.* 2012). As in the case of *S. cerevisiae arv1* cells, the growth defects observed in the presence of the polyenes and papuamide-B suggest that lipid distribution is perturbed at the plasma membrane.

We have previously found, using a disseminated candidiasis mouse model, that *Caarv1/Caarv1* cells were avirulent (Gallo-Ebert *et al.* 2012). In cell culture, *Caarv1/Caarv1* cells had defects in hyphae formation and elongation (Gallo-Ebert *et al.* 2012), and defects in sterol distribution along the growing hyphae (Gallo-Ebert *et al.* 2012), suggesting that CaArv1 regulates sterol distribution, and proper sterol distribution is required for virulence. Uncovering the motifs required for Arv1 function will help to understand the molecular basis for Arv1-driven virulence, while further underscoring the importance of maintaining proper lipid distribution during fungal infection.

Here, we explored the importance of the CaAHD and zinc-binding motifs in CaArv1-dependent virulence. We found that the CaAHD alone cannot replace full-length Arv1 in conferring virulence, while also demonstrating that Cys3 and Cys28 within the zinc-binding motif are essential, as cells expressing full-length *CaArv1^C3S^* or *CaArv1^C28S^* alleles are avirulent. Phenotypically, we found that *Caarv1/Caarv1* cells expressing the CaAHD alone, Caarv1^C3S^, or Caarv1^C28S^ display hyphal formation and elongation defects, as well as sterol distribution defects along the growing hypha. Overall, there was a direct correlation between cells having the ability to maintain proper sterol distribution and virulence, validating our hypothesis that maintaining proper sterol localization is critical for fungal pathogenicity.

## Materials and Methods

### Strain and plasmid construction

Yeast transformations were performed using the Frozen EZ Yeast Transformation II Kit (Zymo Research). All *C. albicans* strains were generated using BWP17 (Table 1). The PCR-based gene disruption method was used for disruption of *CaARV1* (Norice *et al.* 2007). Primers used for cloning are listed in Table 2. Primers (CaARV1-5DR and CaARV1-3DR) were constructed containing 20 bp homologous to the disruption plasmids, p*GEM-URA3* and pRS-*ARG4DSpeI* (Wilson *et al.* 1999), flanked by 70 bp of *CaARV1* sequence, allowing for the replacement of endogenous *CaARV1* with *URA3* and *ARG4*, respectively. Disruption of the endogenous *ARV1* allele was verified by PCR (primers CaARV1-CON5F and CaARV1-CONF3R). All expression plasmids contained 500 bp of the endogenous *CaARV1* promoter, and 500 bp of the *CaARV1* terminator. *CaARV1* (CaARV1-5 COMP and CaARV1-3 COMP), *CaARV1^CgARV1^* (CgARV1-5 COMP and CgARV1-3 COMP), and *CaARV1^AHD^* (CaARV1-AHD-*Bam*HI and CaARV1-AHD-*Sal*I) alleles were generated by PCR. To integrate *CaARV1* alleles into a *Caarv1/Caarv1* homozygous deletion strain, each individual allele was PCR-amplified containing *Not*I sites. Thus, all *Caarv1/Caarv1* strains expressing various *arv1* alleles are heterozygous for each allele. PCR fragments were cloned into the disruption plasmid, pDDB78-*HIS1* (Spreghini *et al.* 2003), which was linearized with *Nru*I, and integrated into the *Caarv1/Caarv1* homozygous deletion strain at the *HIS1* locus. Integration was verified by PCR amplification at the *HIS1* locus (CaHIS-ARV1-DIAG5F, CaHIS-ARV1-DIAG3R, CaHIS-PGEM-DIAG3R, and CaHIS-pDDB78-DIAG3R). Full-length *CaARV1* was used to construct site-directed mutants (CaARV1-C3A-SDM5F, CaARV1-C3A-SDM3R; CaARV1-C28A-SDM5F, CaARV1-C28A-SDM3R; and CaARV1-C28S-SDM5F, CaARV1-C28A-SDM5F). Heterozygous *Caarv1/Caarv1* transformants were selected on synthetic minimal medium lacking uracil, arginine, and histidine, thus obtaining *URA3^+^*, *ARG4^+^*, and *HIS1^+^* transformants. Integration was verified by PCR. All strains were integrated with all selectable markers (*URA3*, *ARG4*, and *HIS1*) to eliminate auxotrophy-specific pleiotrophic effects. All mutant plasmid constructs were sequenced to verify the presence of individual mutations. *q*RT-PCR indicated that there were no copy differences in the expression of the alleles. Point mutations were generated using the QuickChange Site Directed Mutagenesis Kit (Stratagene) and *pDDB78-HIS1-CaARV1* (*pHIS1*) as a template. The endogenous *CaARV1* promoter drove expression of all constructs.

**Table 1:** Strains and genotypes

Strain	Text Designation	Genotype
BWP17		*ARV1 ura3*∆::*limm434*::*URA3 arg4*::*hisG*::*ARG4 his1*::*hisG*
		*ARV1 ura3*∆::*limm434*::*URA3 arg4*::*hisG*::*ARG4 his1*::*hisG*
*ARV1/ARV1 (pHIS1)*	*CaARV1/CaARV1*	*ARV1 ura3*∆::*limm434*::*URA3 arg4*::*hisG*::*ARG4 his1*::*hisG*::*pHIS1*
		*ARV1 ura3*∆::*limm434*::*URA3 arg4*::*hisG*::*ARG4 his1*::*hisG*
*arv1^−^/arv1^−^ (pHIS1-ARV1)*	*Caarv1^−^/CaARV1*	*arv1*::*ARG4 ura3*∆::*limm434 arg4*::*hisG*:: *his1*::*hisG*::*pHIS1-ARV1*
		*arv1*::*URA3 ura3*∆::*limm434 arg4*::*hisG his1*::*hisG*
*arv1^−^/arv1^−^ (pHIS1)*	*Caarv1^−^/Caarv1^−^*	*arv1*::*ARG4 ura3*∆::*limm434 arg4*::*hisG his1*::*hisG*::*pHIS1*
		*arv1*::*URA3 ura3*∆::*limm434 arg4*::*hisG his1*::*hisG*
*arv1^−^/arv1^−^ (pHIS1-ARV1^AHD^)*	*Caarv1^CaAHD^*	*arv1*::*ARG4 ura3*∆::*limm434 arg4*::*hisG his1*::*hisG*::*pHIS1^AHD^*
		*arv1*::*URA3 ura3*∆::*limm434 arg4*::*hisG his1*::*hisG*
*arv1^−^/arv1^−^ pHIS1-ARV1^C3S^)*	*Caarv1^C3S^*	*arv1*::*ARG4 ura3*∆::*limm434 arg4*::*hisG his1*::*hisG*::*pHIS1^C3S^*
		*arv1*::*URA3 ura3*∆::*limm434 arg4*::*hisG his1*::*hisG*
*arv1^−^/arv1^−^ (pHIS1-ARV1^C28S^)*	*Caarv1^C28S^*	*arv1*::*ARG4 ura3*∆::*limm434 arg4*::*hisG his1*::*hisG*::*pHIS1^C28S^*
		*arv1*::*URA3 ura3*∆::*limm434 arg4*::*hisG his1*::*hisG*
*arv1^−^/arv1^−^ (pHIS1-ARV1^CgARV1^)*	*Caarv1^CgARV1^*	*arv1*::*ARG4 ura3*∆::*limm434 arg4*::*hisG his1*::*hisG*::*pHIS1^CgARV1^*
		*arv1*::*URA3 ura3*∆::*limm434 arg4*::*hisG his1*::*hisG*

**Table 2:** Primer sequences

Primer Name	Sequence
CaARV1-5DR (*CaARV1* deletion)	5′-CTGCTCTGATACTAGAGGCATTCAACGCCAGCATGTTTACATTGGGGAAGATACCGGATGTACCACCACTTTCCCAGTCACGACGTT-3′
CaARV1-3DR (*CaARV1* deletion)	5′-AATTGAACACTAAATACGAATACCCCAATCTAGTTAATGATTTAGACGGGCCAATGATTGCATTGGATGGTGTGGAATTGTGAGCGGATA-3′
CaARV1-CON5F (*CaARV1* deletion verification)	5′-GCGAACACCAATCAGAATTCG-3′
CaARV1-CON3R (*CaARV1* deletion verification)	5′-CCTTGAGAGCAATTGAAAGC-3′
CgARV1-CONF2 (*CgARV1* allele integration verification)	5′-CAATATGGGCTCTTCTTCT-3′
CgARV1-CONIR (*CgARV1* allele integration verification)	5′-GCCCATGGTAGGGTGAATACT-3′
CgARV1-5 COMP (*CgARV1* allele generation)	5′-CAAGAATTGGACCATTCCAA-3′
CgARV1-3 COMP (*CgARV1* allele generation)	5′-ACTTTACTTAATGTGATCATCC-3′
CaARV1-5 COMP (*CaARV1* allele generation)	5′-ACACCAATCAGAATTCGTCA-3′
CaARV1-3 COMP (*CaARV1* allele generation)	5′-TTACTGGATTATTGCCAACT-3′
CaARV1-AHD-*Bam*HI (*CaAHD* allele generation)	5′-GCGGATCCCAATCTGCATTTGGAA-3′
CaARV1-AHD-*Sal*I (*CaAHD* allele generation)	5′-GCGTCGACTAATAGTCCCATTCTGAA-3′
CaARV1-C3S-SDM5F (C3S allele generation)	5′-TCCATTTTCAATGATCAGTATAGAATGTGGATATT-3′
CaARV1-C3S-SDM3R (C3S allele generation)	5′-TATCCACATTCTATACTGATCATTGAAAATGGATG-3′
CaHIS-ARV1-DIAG5F (*HIS1* integration verification)	5′-GTTGGTGTGGCCCAGAGAC-3′
CaHIS-ARV1-DIAG3R (*HIS1* integration verification)	5′-GTGACAACTCGTAGTGCCTCC-3′
CaARV1-C28S-SDM5F (C28S allele generation)	5′-TATATCAAACTAAGTGTAAGTCCCGAATGTAATAAAA-3′
CaARV1-C28S-SDM3R (C28S allele generation)	5′-TTTTATTACATTCGGGACTTACACTTAGTTTGATATA-3′
CaHIS-ARV1 DIAG5F (*HIS1* integration verification)	5′-GTTGGTGTGGCCCAGAGAC-3′
CaHIS-ARV1 DIAG3R (*HIS1* integration verification)	5′-GTGACAACTCGTAGTGCCTCC-3′
CaHIS-PGEM-DIAG-3R (*HIS1* integration verification)	5′-CTCCCGGCCGCCATGG-3′
CaHIS-PDDB78-DIAG-3R (*HIS1* integration verification)	5′-TCGAGGTCGACGGTATCGAT-3′

To construct *Caarv1/Caarv1* cells expressing *CgARV1*, the full-length coding sequence of *CgARV1* was subcloned into pDDB78-*HIS1* containing 500 bp of the *CaARV1* promoter, full length *CgARV1* (1000 bp), and 500 bp of the *CaARV1* terminator. *Caarv1/Caarv1* cells expressing *AHD* were generated by integrating *pDDB78-HIS1* containing the *CaARV1* AHD domain.

### Protein isolation and western analysis

Protein extraction was performed as described previously (Villasmil and Nickels 2011). Protein levels were determined using cell lysates and the Bradford assay system (Bio-Rad). Proteins were visualized using immunoblotting and chemoluminescence as described previously (Villasmil and Nickels 2011). Rabbit anti-yeast Arv1 polyclonal antibodies were generated by Lampire Biological Products (Pipersville, PA), and were used at a 1:500 dilution.

### Sterol extraction and analysis

Single colonies of *Candida albicans* strains were grown for 18 hr in YEPD at 37°, 200 rpm; ∼15 ml of culture was harvested and cells were washed twice with ddH_2_O. Cells were then resuspended in 1 ml ddH_2_O and split equally into two tubes, one sample for the determination of the dry weight of cells, the other for sterol extraction.

Nonsaponifiable lipids were prepared and extracted as reported previously (Kelly *et al.* 1995). An internal standard of 10 µg of cholesterol was added prior to extraction with hexane. Samples were dried in a vacuum centrifuge (Heto), and were derivatized by the addition of 100 ml 90% N,O-bis(trimethylsilyl)trifluoroacetamide (BSTFA)/10% trimethylsilyl (TMS) (Sigma) and 200 ml anhydrous pyridine (Sigma), and heating for 2 hr at 80°. TMS-derivatized sterols were analyzed and identified using GC/MS (Agilent 5975C Inert XL GC/MSD) with reference to retention times and fragmentation spectra for known standards. GC/MS data files were analyzed using Agilent software (MSD Enhanced ChemStation, Agilent Technologies, Stockport, UK) to determine integrated peak areas, and enable calculation of the percentage of total sterols and the amount of sterol/dry weight of cells.

### Disseminated candidiasis studies

Female BALB/cJ mice (Jackson Labs) aged 6–8 wk, weighing ∼18–22 g, were housed in groups of as many as four animals, and were supplied food and water *ad libitum*; 8–10 mice were used for each strain. *C. albicans* strains were grown overnight in YEPD medium (1% yeast extract, 2% bactopeptone, and 2% dextrose) at 30°, harvested by centrifugation, washed twice with 1× phosphate-buffered saline (PBS), counted by hemocytometry, and resuspended in 1× PBS at the required density. For survival experiments, mice were injected via the tail vein with 200 µl of 1 × 10^4^ cells/ml of *C. albicans* in 1× PBS. Infected animals were monitored daily for 30 d postinfection, and were considered moribund when they could no longer reach food or water. Moribund animals and mice surviving to the end of the study were killed by CO_2_ asphyxiation, and survival times were recorded. All animals were housed at Temple University—an Association for Assessment and Accreditation of Laboratory Animal Care (AALAC) accredited facility. The Temple University Institutional Animal Care and Use Committee (IACUC) approved the protocol.

For organ fungal load determination, mice were injected via the tail vein with 200 µl of 5 × 10^5^ cells/ml of *C. albicans* in 1× PBS. Animals were killed 48 hr postinfection. Concentrations of yeast inocula were determined by plate viability counts made from organ suspensions. A total of 10 mice/strain was infected for survival and organ fungal load experiments. Experimental procedures were carried out according to the National Institutes of Health (NIH) guidelines for the ethical treatment of animals. Temple University’s IACUC approved all animal use protocols.

### Determination of organ fungal load

Mice infected with *C. albicans* were killed 48 hr postinfection, and target organs (kidney, spleen, and liver) were removed aseptically and homogenized in 4 ml of 1× PBS. Fungal load was determined by making 10-fold serial dilutions in 1× PBS, and plating 40 μl on YEPD plates containing 34 μg/ml chloramphenicol. Plates were incubated at 30° for 24 hr. Total CFUs were determined, and counts were expressed as the log_10_ CFU/organ weight in grams; 8–10 livers were combined and analyzed.

### Chitin staining and fluorescence microscopy

Hyphal formation was induced at 37° for 3 hr in 10% fetal bovine serum (FBS). Cells were fixed with 2% paraformaldehyde for 10 min at room temperature, followed by gentle washing with PBS. Cells were stored at 4° until microscopic analysis. Bud scars were visualized by calcofluor white staining (Sigma-Aldrich, 50–100 μg/ml), with an incubation of 2–5 min at room temperature. Microscopy was performed immediately with 100× magnifications using a Leica fluorescence microscope with an attached camera. At least 300 cells were examined, and the data are the average of five independent experiments.

### Filipin staining and fluorescence microscopy

Unesterified sterol was visualized using filipin staining. One milliliter of 37.5% formaldehyde was added to 9 ml of cell culture grown to a density of 0.7 OD_600_ U/ml. After 10 min of mixing at 23°, fixed cells were centrifuged, and the pellet was washed twice with 10 ml distilled water. Washed cells were resuspended in 1 ml of water; 200 µl was mixed with 4 µl of freshly made 5 mg/ml filipin complex in ethanol (Sigma-Aldrich, St. Louis, MO). After incubating in the dark for 15 min, cells were spotted directly onto slides, and filipin fluorescence was observed with a UV filter set using neutral density filters. For all fluorescence microscopy experiments, samples were mounted on microscope slides, sealed under coverslips with nail polish, and imaged on a Leica fluorescence microscope with an attached camera. Three hundred cells were counted for each strain, and the data are the average of five independent experiments.

### Data availability

Strains and all reagents are available upon request.

## Results

### Caarv1^AHD^, Caarv1^C3S^, and Caarv1^C28S^ strains are avirulent

Expressing the ScAHD alone can restore mating to *Scarv1* cells (Villasmil *et al.* 2011), suggesting it can substitute for full-length ScArv1 function under some circumstances. To determine if the AHD could substitute for full-length ScArv1 in conferring virulence, a *Caarv1/Caarv1* strain was generated expressing a single *Caarv1^AHD^* allele, and it was tested using a murine model of disseminated candidiasis. The percentage survival of *Caarv1^AHD^*-injected mice was compared to those injected with *CaARV1/CaARV1*, *Caarv1/CaARV1*, and *Caarv1/Caarv1* cells.

**Figure 2:**
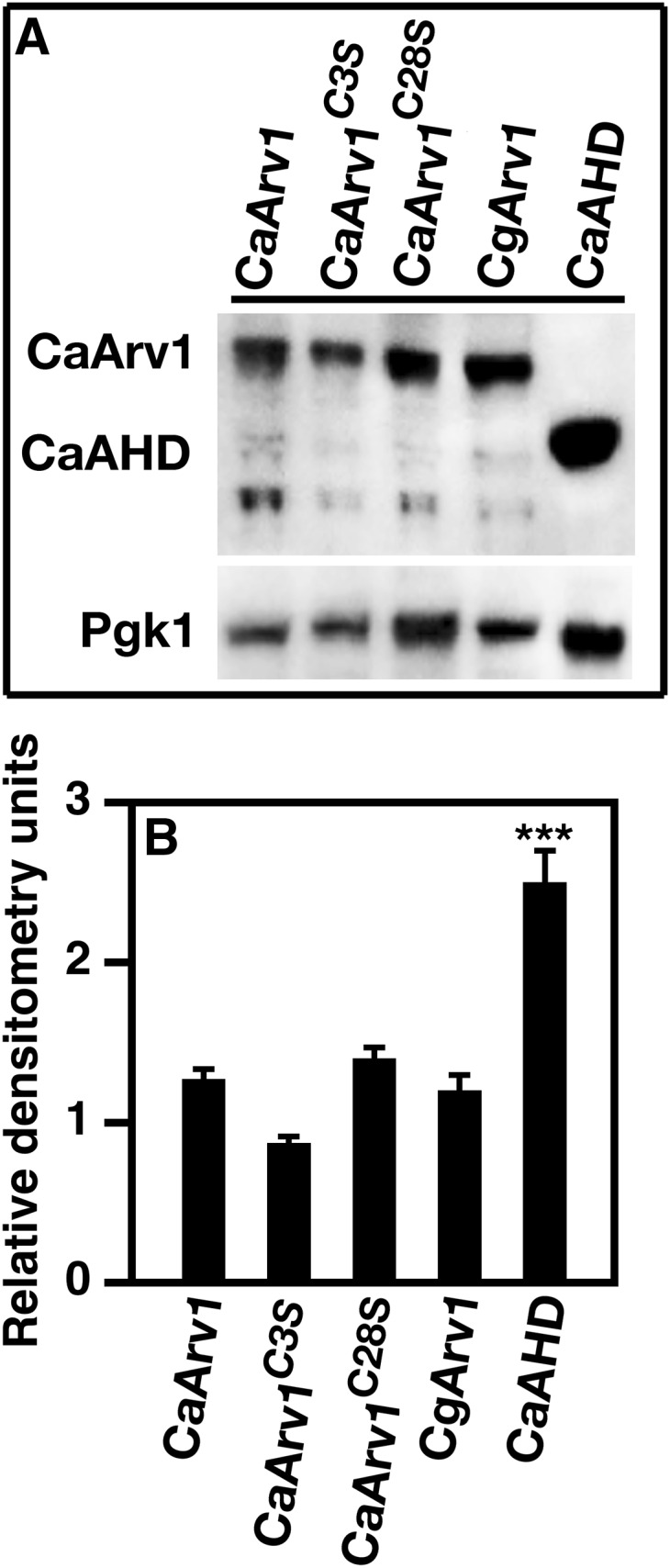
Protein expression levels of various CaArv1 proteins. Cells were grown to exponential phase, then pelleted and cell extracts were obtained. (A) Proteins from cell lysates were resolved by SDS-PAGE. Proteins levels were visualized using immunoblot blot analysis and anti-Ca/ScArv1 polyclonal antibodies. (B) Densitometry of the immunoblot was performed to determine the level of each protein compared to control CaArv1 protein. Densitometry values are the average of five independent experiments. *** *P* < 0.001.

Immunoblot analysis showed that the *Caarv1^AHD^* strain expressed AHD at a level 2.5-fold higher then full-length CaArv1 (Figure 2, A and B). *q*RT-PCR indicated there were no differences in copy numbers (not shown).

**Figure 3:**
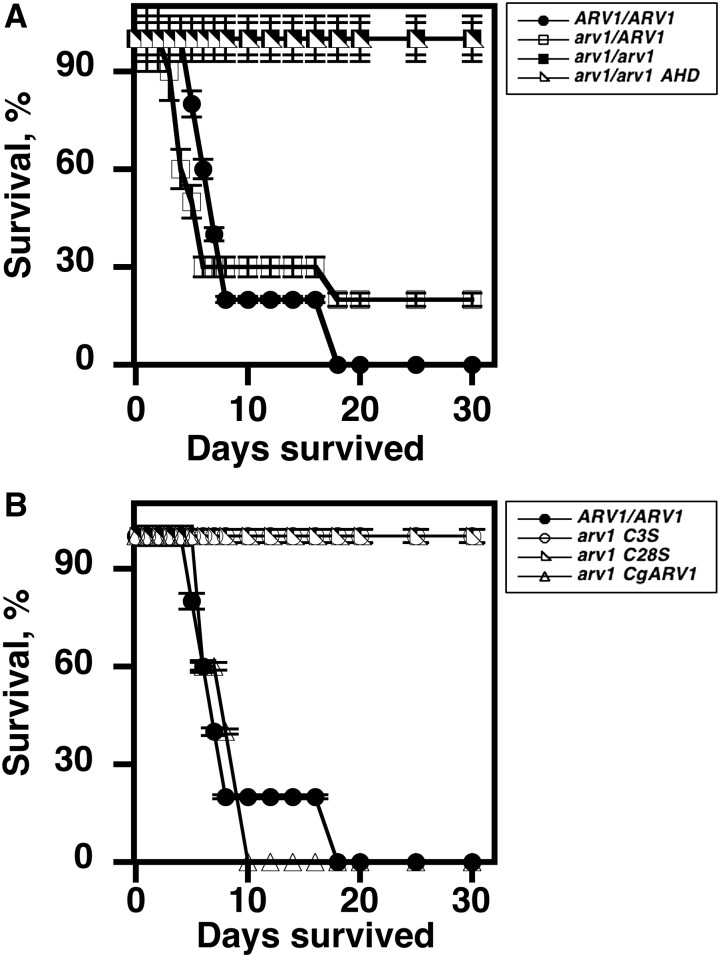
*Caarv1/Caarv1* cells expressing *Caarv1^AHD^*, *Caarv1^3S8^*, or *Caarv1^C28S^* are avirulent. Mice were injected with the strains indicated, and the percentage survival was determined over 30 d. (A) Filled circles, *CaARV1/CaARV1*; open boxes, *Caarv1/CaARV1*; *filled boxes*, *Caarv1/Caarv1*; open triangles, *Caarv1^AHD^*. (B) Open circles, *Caarv1^C3S^*; open triangles, *Caarv1^C28S^*; open pyramids, *Caarv1^CgARV^*.

Mice injected with *CaARV1/CaARV1* cells were dead by d 18, with 50% lost by d 6 (Figure 3A, filled circles), and 50% of mice injected with *Caarv1/CaARV1* cells were dead by d 5 (Figure 3A, open boxes) (*P* < 0.0001) (Table 3). Twenty percent of the remaining mice survived from d 16 to the end of the study, while 100% of mice injected with *Caarv1/Caarv1* cells survived until the study was terminated at 30 d (Figure 3A, filled squares) (*P* < 0.0001). These results are in good agreement with previous work (Gallo-Ebert *et al.* 2012). Mice injected with *Caarv1^AHD^* cells also survived the entire length of the study (Figure 3A, open triangles) (*P* < 0.0001).

**Table 3:** Log rank *P* values

Strain #1	Strain #2	*P*-Value
*CaARV1/CaARV1**^a^*	*Caarv1/CaARV1*	0.96
*CaARV1/CaARV1*	*Caarv1/Caarv1*	<0.0001
*Caarv1/CaARV1**^b^*	*Caarv1/CaARV1^AHD^*	0.0003
*Caarv1/CaARV1*	*Caarv1/CaARV1^C3S^*	<0.0001
*Caarv1/CaARV1*	*Caarv1/CaARV1^C28S^*	0.0003
*Caarv1/CaARV1*	*Caarv1/CaARV1^CgARV1^*	0.85

a Log rank *P* values are compared between the ARV1/ARV1 strain, and the arv1/ARV1 and arv1/arv1 strains.

b Log rank *P* values are compared between the arv1/ARV1 strain and the arv1^AHD^, arv1^C3S^, arv1^C28S^, and arv1^CgARV1^ strains.

Next, we tested the role of the AHD zinc-binding domain in virulence. In this case, amino acids were changed in full-length CaArv1. Cysteines at positions Cys3 (*Caarv1^C3S^*) and Cys28 (*Caarv1^C28S^*) were mutated in the first and second cysteine clusters of the zinc-binding domain (Figure 1, underline). We substituted each Cys with Ser in order to retain tertiary structure (Botello-Morte *et al.* 2016; Stachowiak *et al.* 2009). Immunoblotting analysis showed that CaArv1, CaArv1^C3S^, and CaArv1^C28S^ were expressed equally (Figure 2, A and B). Again, *q*RT-PCR indicated that the copy number of each allele was similar (not shown). Mice injected with either *Caarv1^C3S^* (Figure 3B, open circles) or *Caarv1^C28S^* (Figure 3B, open triangles) cells survived for the length of the study (*P* < 0.0001; *P* < 0.0003).

Finally, we explored the conservation of *ARV1* function by integrating a single *CgARV1* allele into *Caarv1/Caarv1* cells and testing for virulence. Fifty percent of mice injected with *CgARV1^CgARV1^* cells died between d 7 and 8 (Figure 3B, open pyramids) (*P* < 0.05). The remaining mice were dead by d 10.

Our results together indicated that the AHD alone does not possess the same function as full-length CaArv1. They also show that zinc-binding domain function is needed to confer virulence, while indicating a degree of conservation between *CaARV1* and *CgARV1* alleles.

### Abnormal organ fungal loads are seen in mice injected with Caarv1^C3S^, Caarv1^C28S^, and Caarv1^CgARV1^ cells

Multiple tissue failure contributes to the mortality associated with disseminated candidiasis, as organ colonization and invasion is normally seen during an invasive infection (de Repentigny 2004). Thus, fungal loads were determined in the kidney, liver, and spleen, in order to determine if there was any correlation between an increase in organ colonization and virulence.

**Figure 4:**
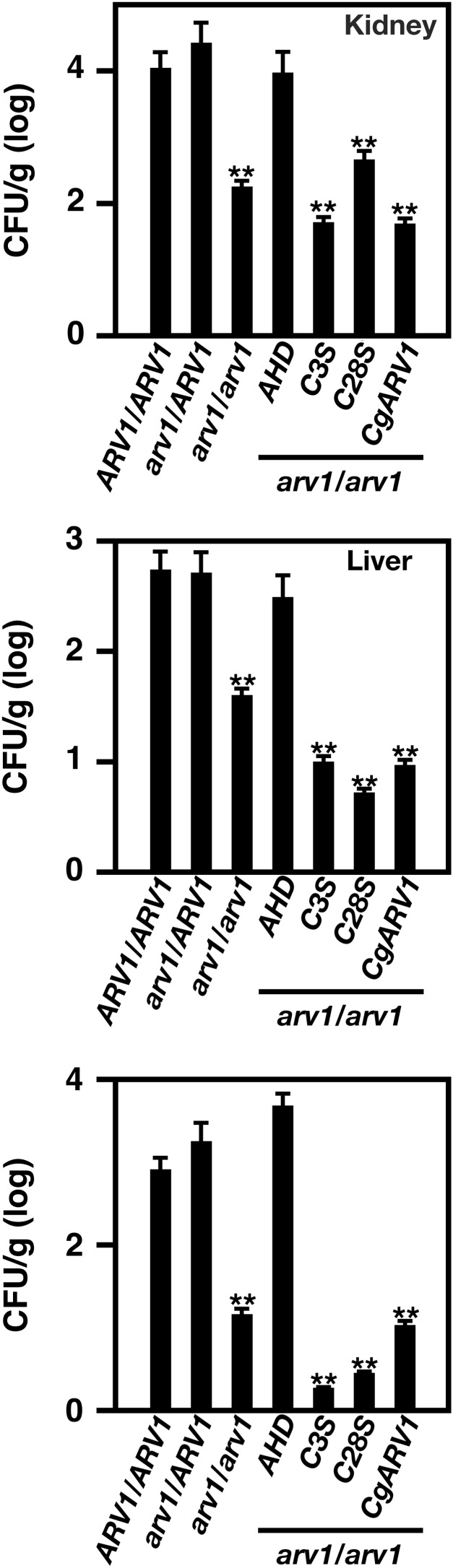
Organ fungal load analysis indicates differences between strains. Mice were injected with 10^5^ cells/ml. Organs were harvested 2 d post injection. Each organ was homogenized and *C. albicans* CFUs were determined by plating homogenates onto YEPD plates containing chloramphenicol. Plates were incubated at 30° for 24 hr. Total CFUs were determined and counts were expressed as the log_10_ CFU/organ weight in grams. The values are the average values obtained from 8 to 10 combined organs from each strain. ** *P* < 0.001.

All organ fungal load levels were similar in *CaARV1/CaARV1­*- and *Caarv1/CaARV1*-injected mice (Figure 4). On the other hand, mice injected with *Caarv1/Caarv1* cells had reduced fungal loads in the kidney, spleen, and liver (Figure 4, *Caarv1/Caarv1 vs. CaARV1/CaARV1*). These results are in good agreement with previous results (Gallo-Ebert *et al.* 2012). The organ fungal loads of *Caarv1^AHD^*-injected mice were similar to that seen for *CaARV1/CaARV1* and *Caarv1/CaARV1* cells, an interesting observation in light of the avirulence of this strain. Mice injected with *Caarv1^C3S^* and *Caarv1^C28S^* cells had reduced fungal loads in all organs. The reduction in fungal load levels in these mutants directly correlates well with the degree of virulence. Unexpectedly, we found that mice injected with *Caarv1^CgARV1^* cells had reduced fungal load levels (Figure 4).

Overall, our results showed that mice injected with *Caarv1^C3S^* and *Caarv1^C28S^* cells had lower fungal loads, and this correlated with increased survival. On the other hand, mice injected with *Caarv1^AHD^* cells, which were avirulent, had normal fungal load levels. Finally, *Caarv1^CgARV1^* cells displayed a higher degree of virulence then did all other cells tested, even though the fungal load levels of mice injected with these cells were drastically lower.

### Avirulent mutant cells expressing Caarv1^AHD^, Caarv1^C3S^, and Caarv1^C28S^ have altered sterol levels

There is strong evidence that Arv1 regulates sterol homeostasis and localization (Gallo-Ebert *et al.* 2012; Georgiev *et al.* 2013; Ruggles *et al.* 2014; Swain *et al.* 2002b; Tinkelenberg *et al.* 2000). *Scarv1* cells accumulate unknown sterols, and a direct correlation exists between accumulation of these intermediates and *Scarv1* phenotypes (Georgiev *et al.* 2013b; Swain *et al.* 2002b; Tinkelenberg *et al.* 2000). Moreover, these cells display sterol distribution defects. *Caarv1/Caarv1* cells are avirulent (Gallo-Ebert *et al.* 2012), and have defects in sterol distribution. To see if there was a correlation between defects in sterol composition and avirulence, sterol intermediates were quantified and their levels were calculated as the percentage of sterol intermediate/total sterol (Table 4).

**Table 4:** Sterol intermediate percentages

Sterol	WT*^a^*	Hetero*^b^*	Null*^c^*	AHD*^d^*	C28S*^e^*	C3S*^f^*	CgARV1*^g^*
Unknown (Ergosta trienol)	0.18 ± 0.02	0.19 ± 0.00	0.17 ± 0.01	0.16 ± 0.02	0.17 ± 0.02	0.25 ± 0.02	0.18 ± 0.01
Ergosta-5,7,22,24(28)-tetraenol	0.34 ± 0.06	0.65 ± 0.10	0.38 ± 0.12	0.28 ± 0.04	0.34 ± 0.12	0.54 ± 0.22	0.60 ± 0.14
Ergosta-5,8,22-trienol	0.43 ± 0.04	0.60 ± 0.10	0.32 ± 0.03	0.43 ± 0.13	0.40 ± 0.01	0.56 ± 0.03	0.53 ± 0.07
Zymosterol	6.0 ± 0.23*^h^*	1.7 ± 0.06*^h^*	1.6 ± 0.30*^h^*	2.1 ± 0.05	2.1 ± 0.09	1.8 ± 0.11	1.7 ± 0.05
Ergosterol (E5,7,22)	73 ± 1.7*^h^*	91 ± 0.45*^h^*	78 ± 0.28	82 ± 0.08	80 ± 1.3	90 ± 0.79	90 ± 1.7
Ergosta-8,22-dienol	0.30 ± 0.08	0.00 ± 0.00	0.00 ± 0.00	0.00 ± 0.00	0.00 ± 0.00	0.00 ± 0.00	0.00 ± 0.00
Ergosta-5,8,22,24(28)-tetraenol	0.59 ± 0.01	0.60 ± 0.09	0.43 ± 0.05	0.56 ± 0.04	0.51 ± 0.05	0.75 ± 0.12	0.70 ± 0.07
Fecosterol (E8,24(28)-trienol)	1.8 ± 0.11*^h^*	0.41 ± 0.02*^h^*	1.1 ± 0.17	0.94 ± 0.09	1.7 ± 0.17*^i^*	3.1 ± 0.10*^i^*	0.58 ± 0.21
Ergosta-5,7,24(28)-trienol	2.6 ± 0.35*^h^*	0.58 ± 0.11*^h^*	2.1 ± 0.20	3.8 ± 0.25	3.8 ± 0.13*^i^*	2.2 ± 0.23*^i^*	1.4 ± 0.25
Ergosta 5,7 dienol	2.4 ± 0.11*^h^*	1.3 ± 0.09	7.1 ± 0.16*^h^*	5.9 ± 0.16*^i^*	5.9 ± 0.1*^i^*	6.0 ± 0.11*^i^*	1.7 ± 0.29
Episterol [E7,24(28)]	4.0 ± 0.23*^h^*	1.0 ± 0.23*^h^*	2.2 ± 0.38	2.1 ± 0.23	2.1 ± 0.15	1.5 ± 0.47	1.7 ± 0.67
Lanosterol/obtusifliol	6.7 ± 1.4*^h^*	0.71 ± 0.06*^h^*	4.8 ± 0.09	1.7 ± 0.08*^i^*	1.7 ± 0.09*^i^*	2.1 ± 0.11*^i^*	0.9 ± 0.21
4-Methyl fecosterol	0.00 ± 0.00	0.37 ± 0.01	0.87 ± 0.13	0.00 ± 0.00	0.50 ± 0.06	0.00 ± 0.00	0.00 ± 0.00
4,4-Dimethylzymosterol	1.6 ± 0.14	1.0 ± 0.17	1.0 ± 0.36	0.18 ± 0.01*^i^*	0.82 ± 0.35	0.14 ± 0.26*^i^*	0.35 ± 0.10
Eburicol	0.12 ± 0.07	0.00 ± 0.00	0.50 ± 0.09	0.18 ± 0.02	0.23 ± 0.06	0.00 ± 0.00	0.00 ± 0.00

a ARV1/ARV1.

b arv1/ARV1.

c arv1/arv1.

d arv1^AHD^.

e arv1^C28S^.

f arv1^C3S^.

g CgARV1.

h The percentage differences between ARV1/ARV1 and arv1/ARV, and arv1/arv1.

i The percentage differences between arv1/ARV1 and arv1^AHD^, arv1^C3S^, arv1^C28S^, and arv1^CgARV1^.

Interestingly, the sterol compositions of *CaARV1/CaARV1* and *Caarv1/CARV1* cells were different (Table 4, WT *vs.* hetero). Heterozygous cells had a higher percentage of ergosterol (130%), and decreased percentages of zymosterol (28%), episterol (25%), fecosterol (23%), ergosta-5,7,24(28)-trienol (23%), and lanosterol (10%) compared to *CaARV1/CaARV1* cells (Table 4). *Caarv1/Carv1* cells had a higher percentage of ergosta 5,7 dienol (300%), and a lower percentage of zymosterol (26%) compared to *CaARV1/CaARV1* cells.

To next examine if the AHD, Cys3, and Cys28 were required for maintaining normal sterol composition, sterol content was determined in cells expressing *Caarv1^AHD^*, *Caarv1^C3S^*, or *Caarv1^C28S^* alleles. Sterol intermediates levels were compared to *Caarv1/CaARV1* cells. *Carv1^AHD^* cells had a higher percentage of ergosta 5,7 dienol (246%) and a lower percentage of 4,4-dimethylzymosterol (10%), whereas both *Carv1^C3S^* and *Carv1^C28S^* cells had higher percentages of fecosterol (415%), ergosta-5,7,24(28)-trienol (630%), ergosta 5,7 dienol (454%), and lanosterol (242%). The sterol composition of *Carv1^CgARV1^* cells was similar to that of *Caarv1/CaARV1* cells.

In looking at the data as a whole, it is interesting that all avirulent strains accumulated the same sterol intermediate, ergosta 5,7 dienol: [*Caarv1/Carv1* (300%), *Caarv1^AHD^* (246%), *Carv1^C3S^* (630%), and *Carv1^C28S^* (630%)].

### Proper hyphal formation is delayed in avirulent strains

**Figure 5:**
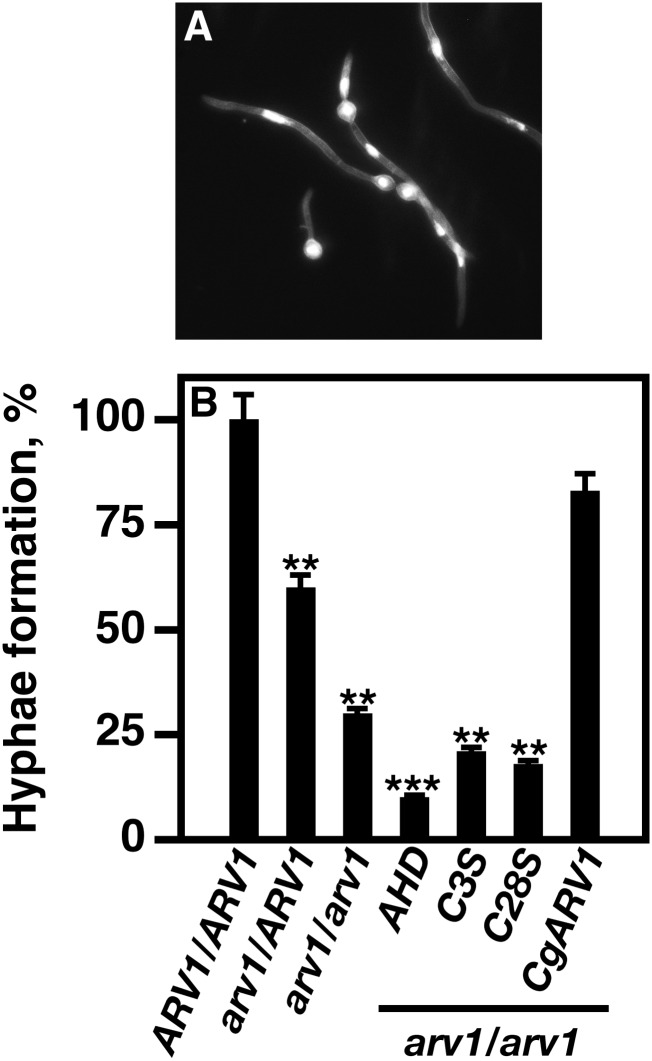
Hyphal formation is delayed in strains carrying Caarv1^AHD^, *Caarv1^C3S^*, and *Caarv1^C28S^* alleles. Various *Caarv1* strains were grown to exponential phase in YEPD at 30°. Invasive growth was initiated by shifting cultures to 37° for 3 hr in 10% FBS. Hyphal formation was determined at 3 hr using light microscopy. (A) DAPI stained *CaARV1/CaARV1* cells. (B) Percentage of cells forming hyphae. Percentages are the average of five independent experiments. ** *P* < 0.001; *** *P* < 0.0001.

Hyphal formation and subsequent elongation are necessary for strains to be virulent (Lu *et al.* 2014). In order to understand the molecular basis underlying avirulence, hyphal initiation and formation were visualized in cell culture using fluorescence microscopy (Figure 5A). Cells were visualized at 3 hr after growth in invasive medium.

Interestingly, *Caarv1/CaARV1* cells did have a reduction in the number of cells forming hyphae compared to *CaARV1/CaARV1* cells (Figure 5B, *P* < 0.001). The percentages of *Caarv1/Caarv1*, *Caarv1^AHD^*, *Caarv1^C3S^*, and *Caarv1^C28S^* cells forming hyphae were also significantly reduced (Figure 5B, *P* < 0.0001; *P* < 0.001; *P* < 0.001, respectively). *Caarv1/Caarv1* cells expressing *CgARV1* did not display a reduction in hyphal formation.

### Avirulent cells have defects in bud site selection and septa formation

Bud site selection along the mother cell periphery dictates where hyphal formation will initiate (Lu *et al.* 2014). The initial step of hyphal biogenesis is the formation of the germ tube, which emerges in a predominately nonaxial position (bipolar or random). Hyphal branches then emerge adjacent to locations of hyphal septa, on the mother (proximal) side (Gow and Hube 2012; Hausauer *et al.* 2005). To further our understanding of why mutant cells were delayed in hyphal formation, we visualized septa number and location, and the positioning of bud sites using calcofluor white and fluorescence microscopy. Chitin localization was visualized *in vitro* after cells were grown in hyphae-inducing medium for 3 hr.

**Figure 6:**
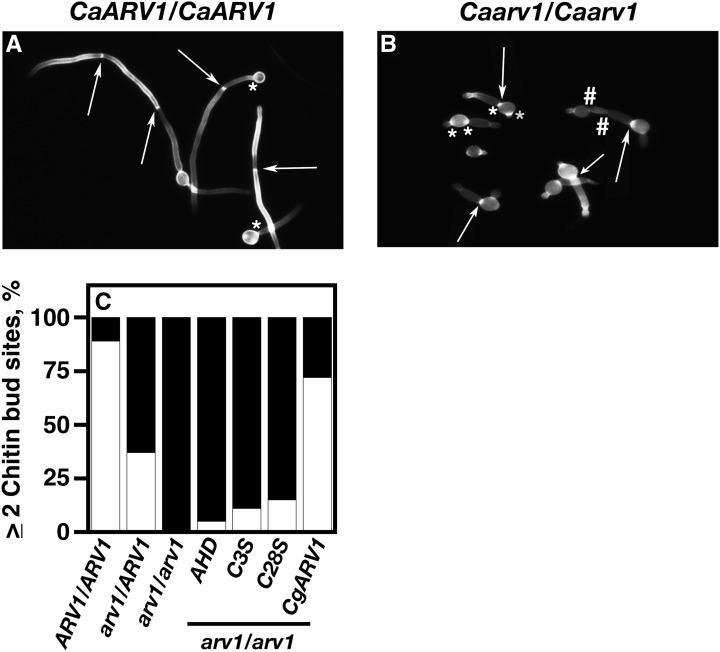
Bud site selection and septa formation are defective in strains carrying *Caarv1^AHD^*, *Caarv1^C3S^*, and *Caarv1^C28S^* alleles. Various *Caarv1* strains were grown to exponential phase in YEPD at 30°. Invasive growth was initiated by shifting cultures to 37° for 3 hr in 10% FBS. (A, B), Bud site selection was determined at 3 hr by fixing cells in paraformaldehyde and staining with calcoflour white. (A) *CaARV1/CaARV1* cells (arrows, septa; asterisk, chitin stained bud site). (B) *Caarv1/Caarv1* cells (arrows, chitin stained bud site; hash signs, constrictions along the hyphae; asterisk, cells with two chitin stained bud sites). (C) White bars, number of cells with ≥2 chitin bud sites; black bars, number of cells with < 2 chitin bud sites.

Examples of septa (Figure 6A, arrows) and chitin bud site staining (Figure 6B, asterisk) are shown posthyphal initiation for *CaARV1/CaARV1* and *Caarv1/Caarv1* cells, respectively. When examined, *CaARV1/CaARV1* cells had ≥2 septa (Figure 6A, arrows) along a single hypha; ∼85% of *CaARV1/CaARV1* cells initiated hypha from a single mother–daughter chitin bud site at 3 hr postinitiation (Figure 6A, asterisk). The number of *Caarv1/CaARV1* cells having ≥ 2 septa was reduced to ∼30% of that seen in *CaARV1/CaARV1* cells (Figure 6C); however, *Caarv1/CaARV1* cells were normal for septa formation, and for the number and positioning of chitin bud sites. *Caarv1/Caarv1* mutants had a reduction in the numbers of hyphae formed compared to *CaARV1/CaARV1* cells (Figure 6C, ∼65%, *P* < 0.001). These mutants had constrictions along the germ tube, which lacked chitin staining (Figure 6B, hash sign), and hyphal initiation was initiated from a single bud site. Another interesting phenotype displayed by *Caarv1/Caarv1* cells was that they had a second chitin bud site that was the initiating point for another germ tube (Figure 6B, asterisk). A high percentage of *Caarv1^AHD^* (∼95%), *Caarv1^C3S^* (90%), and *Caarv1^C28S^* (85%) cells had only a single chitin bud site that remained at the initial mother–daughter neck (Figure 6C, black bars), and ∼75% of *Caarv1^CgARV1^* cells had a single chitin bud site and ≥ 2 septa along a single hypha (Figure 6C, black bars).

### Avirulence correlates directly with defects in ergosterol distribution

The loss of *S. cerevisiae* Arv1 causes sterol distribution defects (Georgiev *et al.* 2013; Villasmil *et al.* 2011). There is a direct correlation between the degree of sterol defects and a reduction in mating efficiency (Villasmil *et al.* 2011). Sterol distribution defects are also seen in *Caarv1/Caarv1* cells, and severity correlates directly with loss of hyphal formation and the degree of avirulence (Gallo-Ebert *et al.* 2012). Thus, there is a relationship between loss of Arv1 function, defects in sterol distribution, and signaling-dependent polarized growth.

To see if there was a correlation between lack of sterol localization and avirulence, the localization of cellular sterol was visualized using filipin staining and fluorescence microscopy.

Qualitatively, we found that all cells took up the same level of filipin, so we reasoned that any defects observed would not be due to lack of dye internalization.

**Figure 7:**
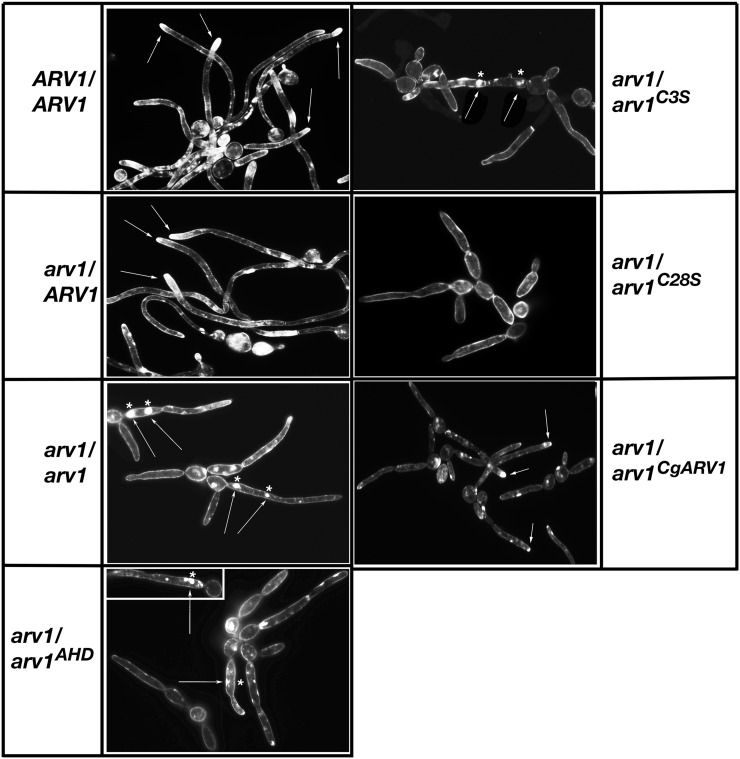
Sterol distribution during hyphal development is defective in strains carrying Caarv1^AHD^, *Caarv1^C3S^*, and *Caarv1^C28S^* alleles. Various *Caarv1* strains were grown to exponential phase in YEPD at 30°. Invasive growth was initiated by shifting cultures to 37° for 3 hr in 10% FBS. Sterol localization was determined at 3 hr by fixing cells in paraformaldehyde and staining with filipin. Sterol localization was visualized by fluorescence microscopy using a Leica DRME microscope. Arrows indicate sterol localization during hyphal growth; asterisks indicate defective sterol localization.

**Figure 8:**
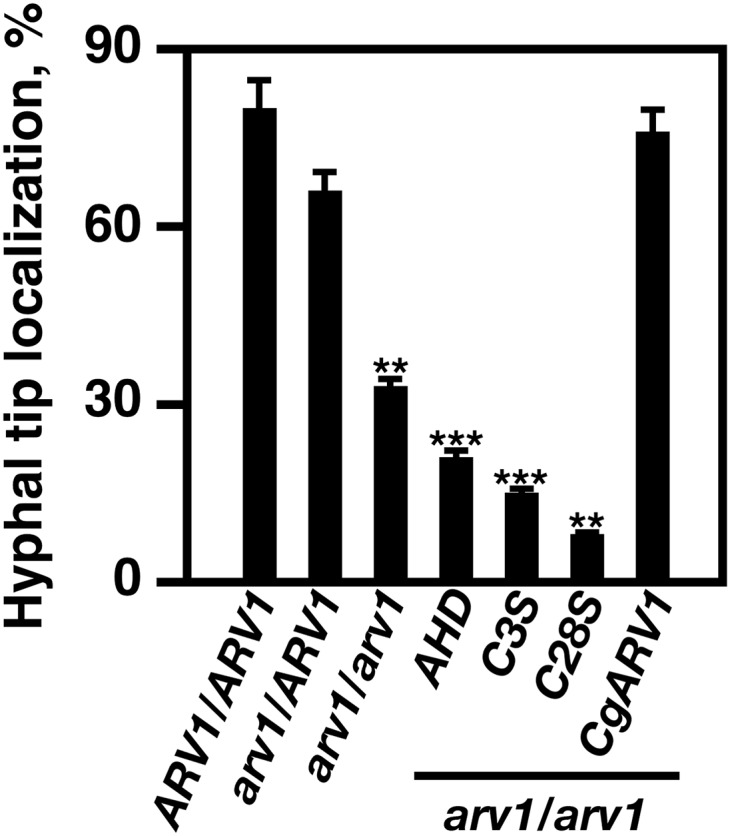
Sterol localization to the hyphal tip is defective in Caarv1^AHD^, *Caarv1^C3S^*, and *Caarv1^C28S^* allele expressing strains. *Caarv1* strains were grown to exponential phase in YEPD at 30°. Invasive growth was initiated by shifting cultures to 37° for 3 hr in 10% FBS. Sterol localization was determined at 3 hr was determined by fixing cells in paraformaldehyde and using filipin staining and fluorescence microscopy. Sterol localization was visualized using a Leica DRME microscope. The data are the average of five independent experiments. ** *P* < 0.001; *** *P* < 0.0001.

*CaARV1/CaARV1* and *Caarv1/CaARV1* cells had a similar percentage of cells having normal distribution (Figure 7), localizing the majority of their sterol to the growing hyphal tip (Figure 8, *arrows*). *Caarv1*/*Caarv1*, *Caarv1^AHD^*, *Caarv1/Caarv1^C3S^*, and *Caarv1/Caarv1^C28S^* cells all showed defects in sterol distribution (Figure 7). The percentage of *Caarv1*/*Caarv1* cells properly localizing their sterol was reduced to ∼30% of that seen for *Caarv1/CaARV1* cells (Figure 8). *Caarv1*/*Caarv1* cells accumulated large sterol aggregates that were localized centrally (Figure 7, *Caarv1/Caarv1*; arrows and asterisks). The percentage of *Caarv1^AHD^* cells with hyphal tip-localized sterol was lower than that seen for *Caarv1/Caarv1* cells (20%) (Figure 8). *Caarv1^AHD^* cells accumulated aggregates that were situated more at the cell periphery (Figure 7, *Caarv1^AHD^*; arrows and asterisks). *Caarv1*/*Caarv1^C3S^* and *Caarv1^C28S^* cells had the least number of cells localizing their sterol to the hyphal tip (Figure 8, ∼10%). They both accumulated sterol aggregates and had a diffuse sterol localization concentrated at the plasma membrane surface (Figure 7). Finally, *Caarv1^CgARV1^* cells properly distributed and localized their sterol (Figure 7 and Figure 8, *Caarv1^CgARV1^*).

Thus, *Caarv1*/*Caarv1*, *Caarv1^AHD^*, *Caarv1/Caarv1^C3S^*, and *Caarv1/Caarv1^C28S^* cells lack virulence. These strains also had sterol distribution defects and accumulated what appeared to be sterol aggregates. Thus, we can conclude that there was a direct correlation between cells being avirulent and their lack of ability to properly distribute their sterol during hyphal growth.

## Discussion

*C. albicans* strains lacking CaArv1 are avirulent, suggesting that Arv1 function has a role in maintaining virulence. CaArv1 contains a CaAHD domain that has within it a zinc-binding motif. Here, we explored whether the CaAHD alone was responsible for the virulence function of CaArv1, and, if so, was the zinc-binding motif necessary for virulence. The CaAHD alone could not replace full-length Arv1 function, suggesting that additional domains outside the AHD play a role in virulence. However, we did find that the CaAHD zinc-binding motif was needed for virulence, as cells containing an intact CaArv1 protein harboring either a Cys3 or Cys28 mutation were avirulent, substantiating the hypothesis that CaAHD function is necessary for virulence but is not sufficient. These data hint at the possibility that the activity of the zinc-binding motif is the critical function associated with CaAHD.

There was a strong association between how virulent a strain was and its ability or inability to distribute sterol. Avirulent *Caarv1^AHD^*, *Caarv1^C3S^*, and *Caarv1^C28S^* cells all had defects in sterol distribution and septa formation, and all lacked the ability to localize their sterol to the growing hyphal tip. Interestingly, these mutant strains accumulated several sterol biosynthetic intermediates when compared to wild-type cells. *S. cerevisae* cells lacking ScArv1 have elevated sterol and phosphatidylserine levels in their plasma membrane (Georgiev *et al.* 2013; Tinkelenberg *et al.* 2000), display sterol distribution defects during mating (Villasmil *et al.* 2011), are unable to mobilize PIP_2_ (Villasmil *et al.* 2011), and accumulate unknown sterol intermediates (Swain *et al.* 2002a). The results in *S. cerevisiae*, along with those presented here, lend strong support to the theory that CaArv1 is highly conserved, and that it regulates sterol distribution during *C. albicans* invasion. Just as important, it also strongly suggests that maintaining sterol distribution is critical for *C. albicans* infection.

Organ colonization and invasion are considered major mortality factors, especially in the case of the kidney (Ashman *et al.* 1996; Fisher *et al.* 2011; Vecchiarelli *et al.* 1988). Mice infected with the *C. albicans arv1^AHD^* strain had normal kidney fungal loads, but survived for the entire length of the study. There are several avirulent *C. albicans* mutants that cause elevated kidney CFUs (Douglas *et al.* 2009; Epp *et al.* 2010), so there is precedence for this observation. On the other hand, mice injected with either *Caarv1^C3S^* or *Caarv1^C28S^* cells had reduced fungal loads, and this correlated well with avirulence, suggesting that the zinc-binding motif has a role in organ colonization and invasion. Unexpectedly, we found that mice injected with *Caarv1^CgARV1^* had reduced organ fungal loads, suggesting an increase in fungal clearance. The reason for this phenotype is unclear to us. One possibility is that the *Caarv1^CgARV1^* strain acts as a superantigen, causing a rapid response that causes early organ failure. Animals infected with *Caarv1^CgARV1^* do die much sooner than those infected with other virulent strains. Thus, we may have missed the most appropriate time to demonstrate colonization and invasion. Superantigen effects have been seen during *S. pneumonia* infection (Tilahun *et al.* 2014), initiation of toxic shock (Hanna and Tierno 1985; Meedt *et al.* 2010), and *Staphylococcus aureus* infection (Langley *et al.* 2010). It is interesting to point out that *Caarv1^CgARV1^* cells secrete higher levels of aspartyl proteases (P. McCourt, unpublished data). Whether this increased secretion contributes to increased pathogenicity is presently being explored.

*Caarv1/Caarv1* and *Caarv1/Caarv1^AHD^* cells were delayed in forming hyphae, accumulated large sterol aggregates, and had a reduced number of cells localizing their sterol to the hyphal tip. On the other hand, *Caarv1^C3S^* and *Caarv1^C28S^* cells had a diffuse peripheral sterol-staining pattern, but were also delayed in hyphal formation. *S. cerevisiae* mating haploids must localize their sterol to the polarized mating projection tip in order to mate (Bagnat and Simons 2002; Jin *et al.* 2008; Proszynski *et al.* 2006; Simons and Toomre 2000; Villasmil *et al.* 2011). *Scarv1* cells are sterile, and this correlates with sterol distribution defects and a reduction in mating projection formation (Villasmil *et al.* 2011). *C. albicans* cells localize their sterol to cell septa and hyphal tips upon initiating invasive growth (Gallo-Ebert *et al.* 2012; Martin and Konopka 2004), and this is required for hyphal formation (Chen and Thorner 2007; Sudbery 2011). Thus, both ScArv1 and CaArv1 seem to distribute sterol to sites of membrane clustering and polarization.

Data suggest that Arv1 has the ability to distribute lipids other than sterol, including the glycerophospholipid, phosphatidylserine. *Scarv1* mutants are hypersensitive to the phosphatidylserine-binding agent, papuamide B, suggesting a mislocalization of this lipid to the outer plasma membrane. Studies have shown that phosphatidylserine flipping is required for mating projection formation in *S. cerevisiae*, indicating that phosphatidylserine must be properly localized for maintaining polarized growth (Sartorel *et al.* 2015). Interestingly, *Scarv1* cells have defects in localizing factors required for phosphatidylserine distribution and polarized growth. Scs2 is required for phosphatidylserine transport, and its loss causes phosphatidylserine transport defects, abnormal bud morphology, and sporulation defects (Riekhof *et al.* 2014). *Scarv1* cells cannot properly localize the C-terminal portion of Scs2 to the endoplasmic reticulum. The *C. albicans* ORF 19.1212 is orthologous to Scs2 (http://www.candidagenome.org/cgi-bin/locus.pl?locus=C6_04100W_B). The orf 19.1212 protein product has a FFAT domain (Hanada *et al.* 2009), and is proposed to be a lipid transporter. Whether Scs2 is involved in mating, and if 19.1212 is involved in virulence and/or regulates lipid distribution during invasion, remains to be studied. Interestingly, the phosphatidylserine synthase Cho1 and the phosphatidylserine decarboxylase Psd1 have been shown to be required for filamentous growth in *S. cerevisiae* and virulence in *C. albicans* (Chen *et al.* 2010). Thus, there exists a link between Arv1 function, maintaining proper phosphatidylserine homeostasis, and fungal infection.

Overall, our data strongly suggest that multiple domains of *C. albicans* Arv1 are required for function and virulence. They also indicate that the CaAHD is necessary for virulence, but it alone cannot substitute for full-length CaArv1. Moreover, we have validated the importance of the zinc-binding domain in conferring virulence. AHD homology searches indicate that the AHD and zinc-binding domain are conserved among a large population of pathogenic yeasts. Thus, targeting Arv1 for drug discovery may represent a novel approach for treating systemic candidiasis.
